# Supplementary material for: Multi‐dataset identification of innovative feature genes and molecular mechanisms in keratoconus
Source: J Cell Mol Med. 2024 Sep 19;28(18):e70079. doi: 10.1111/jcmm.70079 (PMC11412914; doi:10.1111/jcmm.70079)
Supplement: Supplementary file 2 — Material S2. [file JCMM-28-e70079-s002.zip › 7.GSEA analysis/5.RNF217-AS1/KEGG_BASAL_TRANSCRIPTION_FACTORS.html]

Details for gene set KEGG\_BASAL\_TRANSCRIPTION\_FACTORS[GSEA]

|  || Dataset | RNF217-AS1.group.cls#h\_versus\_l |
| Phenotype | group.cls#h\_versus\_l |
| Upregulated in class | l |
| GeneSet | KEGG\_BASAL\_TRANSCRIPTION\_FACTORS |
| Enrichment Score (ES) | -0.32285926 |
| Normalized Enrichment Score (NES) | -1.3771169 |
| Nominal p-value | 0.14880952 |
| FDR q-value | 0.3650045 |
| FWER p-Value | 0.889 |
Table: GSEA Results Summary

  

Fig 1: Enrichment plot: KEGG\_BASAL\_TRANSCRIPTION\_FACTORS      
 Profile of the Running ES Score & Positions of GeneSet Members on the Rank Ordered List

  

| SYMBOL | TITLE | RANK IN GENE LIST | RANK METRIC SCORE | RUNNING ES | CORE ENRICHMENT || 1 | TBPL2 | na | 4999 | 0.217 | -0.0649 | No |
| 2 | GTF2A1 | na | 8932 | 0.107 | -0.1393 | No |
| 3 | TAF13 | na | 9687 | 0.092 | -0.1275 | No |
| 4 | TAF1 | na | 11022 | 0.070 | -0.1405 | No |
| 5 | GTF2E2 | na | 12137 | 0.054 | -0.1531 | No |
| 6 | GTF2IRD1 | na | 12800 | 0.045 | -0.1558 | No |
| 7 | GTF2H1 | na | 12997 | 0.042 | -0.1460 | No |
| 8 | TAF5L | na | 13609 | 0.035 | -0.1509 | No |
| 9 | GTF2I | na | 15760 | 0.011 | -0.2088 | No |
| 10 | GTF2F2 | na | 16266 | 0.006 | -0.2211 | No |
| 11 | TAF6L | na | 16806 | 0.001 | -0.2365 | No |
| 12 | GTF2F1 | na | 17848 | -0.009 | -0.2630 | No |
| 13 | TAF12 | na | 19358 | -0.024 | -0.2978 | No |
| 14 | TAF2 | na | 19385 | -0.024 | -0.2898 | No |
| 15 | TBPL1 | na | 20414 | -0.034 | -0.3069 | No |
| 16 | TAF7 | na | 20967 | -0.040 | -0.3084 | Yes |
| 17 | GTF2A2 | na | 21099 | -0.041 | -0.2972 | Yes |
| 18 | GTF2B | na | 21104 | -0.041 | -0.2823 | Yes |
| 19 | GTF2E1 | na | 22078 | -0.052 | -0.2915 | Yes |
| 20 | TBP | na | 22218 | -0.053 | -0.2761 | Yes |
| 21 | TAF10 | na | 22248 | -0.053 | -0.2575 | Yes |
| 22 | TAF1L | na | 22358 | -0.055 | -0.2407 | Yes |
| 23 | TAF9B | na | 22687 | -0.058 | -0.2289 | Yes |
| 24 | GTF2H3 | na | 22951 | -0.061 | -0.2141 | Yes |
| 25 | TAF6 | na | 23046 | -0.062 | -0.1940 | Yes |
| 26 | TAF9 | na | 23072 | -0.063 | -0.1718 | Yes |
| 27 | TAF11 | na | 23261 | -0.065 | -0.1536 | Yes |
| 28 | TAF4 | na | 23472 | -0.068 | -0.1349 | Yes |
| 29 | GTF2H4 | na | 27438 | -0.135 | -0.1998 | Yes |
| 30 | STON1 | na | 27602 | -0.139 | -0.1538 | Yes |
| 31 | TAF4B | na | 28585 | -0.165 | -0.1219 | Yes |
| 32 | TAF5 | na | 31100 | -0.246 | -0.1046 | Yes |
| 33 | TAF7L | na | 34340 | -0.573 | 0.0112 | Yes |
Table: GSEA details [plain text format]

  

Fig 2: KEGG\_BASAL\_TRANSCRIPTION\_FACTORS      
 Blue-Pink O' Gram in the Space of the Analyzed GeneSet

  

Fig 3: KEGG\_BASAL\_TRANSCRIPTION\_FACTORS: Random ES distribution      
 Gene set null distribution of ES for **KEGG\_BASAL\_TRANSCRIPTION\_FACTORS**
